# Inter-departmental variation in surgical treatment of proximal femoral fractures: A nationwide observational cohort study

**DOI:** 10.1371/journal.pone.0281592

**Published:** 2023-02-09

**Authors:** Björn Hernefalk, Emilia Möller Rydberg, Jan Ekelund, Cecilia Rogmark, Michael Möller, Nils P. Hailer, Sebastian Mukka, Olof Wolf

**Affiliations:** 1 Department of Surgical Sciences, Orthopaedics, Uppsala University, Uppsala, Sweden; 2 Institute of Clinical Sciences, Sahlgrenska Academy, Gothenburg University, Gothenburg, Sweden; 3 Centre of Registers, Västra Götaland, Gothenburg, Sweden; 4 Department of Orthopaedics, Lund University, Skåne University Hospital, Malmö, Sweden; 5 Department of Surgical and Perioperative Sciences (Orthopaedics), Umeå University, Umeå, Sweden; Baqai Medical University, PAKISTAN

## Abstract

**Background and purpose:**

Hip fractures should be treated based on the best available evidence and cost-effectively to optimize the outcome for this large group of frail patients. This study examined nationwide variation in surgical methods used for hip fractures.

**Methods:**

In this cohort study 46,243 patients ≥65 years with a trochanteric hip fracture (THF) or a femoral neck fracture (FNF) registered in the Swedish Fracture Register (SFR) between 1 January 2016 and 31 December 2020 were included. Fractures were classified according to the AO Foundation/Orthopaedic Trauma Association (AO/OTA) fracture classification system. The choice of surgical methods was assessed for each fracture type to compare national variation.

**Results:**

21,312 THFs and 24,072 FNFs (67% women) with a mean age of 83 years (SD 8) were surgically treated. In the treatment of two-fragment THFs (AO/OTA A1) departments ranged from using 90% short intramedullary nails to 98% sliding hip screws. Treating displaced FNFs (AO/OTA B3), the proportion of hemiarthroplasty ranged from 9 to 90%, and internal fixation between 0.6 to 21%, depending on the department.

**Interpretation:**

A mature national fracture register permits the monitoring of treatment provided and thus serves as an important aid in assessing compliance with guidelines. The large inter-departmental variation in the surgical management of hip fractures in Sweden appears unwarranted based on the current evidence, indicating a need for updated national guidelines. Further research will have to clarify the impact of this variation on mortality and re-operation rates.

## Introduction

### Background

Although many countries have national guidelines on hip fracture treatment, adherence to these guidelines is hard to monitor. Also, the treating surgeon is often marked by personal preferences based on education, experience and local tradition. A mature national quality register aims to monitor treatment methods and their outcome.

In recent years several organisations, such as the National Institute for Health and Care Excellence (NICE) in Great Britain and the American Association for Orthopaedic Surgeons (AAOS), have published national guidelines for the management of hip fractures [1, 2]. Such policies support the ambition of providing up-to-date, high-quality, evidence-based, prioritised and consistent management of these injuries. Implementing guidelines in conjunction with enhanced renumeration (“Best Practice Tariff”) has been reported to have a positive effect on patient survival and patient-reported outcomes [3, 4].

For trochanteric hip fractures (THFs), the recommendations are to use extramedullary implants, such as sliding hip screws (SHSs) [1]) or intramedullary nails (IMNs) or SHSs [2]. For displaced FNFs in older people, the recommendation is to treat these fractures with cemented arthroplasty [1, 2]. Total hip arthroplasty (THA), is favored over hemiarthroplasty (HA), in active, independent walkers without cognitive impairment [1].

In treating THFs available evidence does not favor a specific implant [5]. IMNs may be associated with more post-operative complications and slightly higher 30-day mortality [6, 7]. Even considering this evidence, the use of IMNs is steadily increasing in many countries, even against the recommendations of national guidelines and a greater cost burden on health care [8, 9].

For displaced femoral neck fractures (FNFs) in the elderly, the treatment strategy in Scandinavia has gradually moved from internal fixation (IF), hoping to preserve the native hip, to primary hip replacement with findings of better function and fewer re-operations [10, 11]. Additionally, primary replacement is a cost-effective treatment for displaced FNFs compared to IF during the first 2 years after fracture [12]. The preservation of the native hip in undisplaced FNFs in older people has been questioned, with findings of higher re-operation rates after IF compared to primary arthroplasty [13]. In line with this observation the 2022 annual report from the Australian and New Zealand Hip fracture Registry reported an arthroplasty frequency of approximately 60% for these fractures in 2021 [14].

In most patients current evidence cannot detect any differences of clinical importance between THA and HA approaches [15]. Notable is the high age cut-off (60 to 70 years) [16, 17] in Scandinavia for using IF for displaced FNFs. Thus, many healthy “young old” will undergo IF rather than (total) arthroplasty.

Many countries still lack national guidelines for managing hip fractures, which might confer worse outcomes for mortality, risk of re-operation and function in patients not treated according to current best practice. Swedish guidelines have not been updated since 2003 [18].

### Objective

The present study aimed to examine nationwide variation by department in the treatment of hip fractures in Sweden stratified by fracture type.

## Patients and methods

### Study design and setting

This observational study was based on data from the Swedish Fracture Register (SFR), which contains data on injury mechanism, fracture classification and treatment (operative and nonoperative) of Swedish citizens with a fracture sustained in Sweden [19]. Proximal femoral fractures are classified according to the AO/OTA classification (2007 version) [20]. This classification, performed by the treating surgeon, is highly accurate and valid compared to an expert group [21]. The coverage of the SFR has increased because of a stepwise introduction from one active department in 2011, 75% coverage in 2016 (41 of 54 departments) to full national coverage in 2021. Based on data from the Swedish National Patient Register, the completeness of femoral fracture registrations in the SFR was 81% in 2020.

THFs are classified into stable two-fragment (AO/OTA **A1**), unstable multi-fragmentary (AO/OTA **A2**) and reverse oblique/subtrochanteric fractures (AO/OTA **A3**). FNFs are classified into non-displaced or minimally displaced (AO/OTA **B1**), basicervical (AO/OTA **B2**) and displaced (AO/OTA **B3**). Treatment is registered as operative or nonoperative. Operative treatment is divided into IF by pins, screws, plates, SHS, IMN or arthroplasty by cemented/uncemented hemi- or total hip arthroplasty techniques.

### Patients and outcome measures

**Fig 1:**
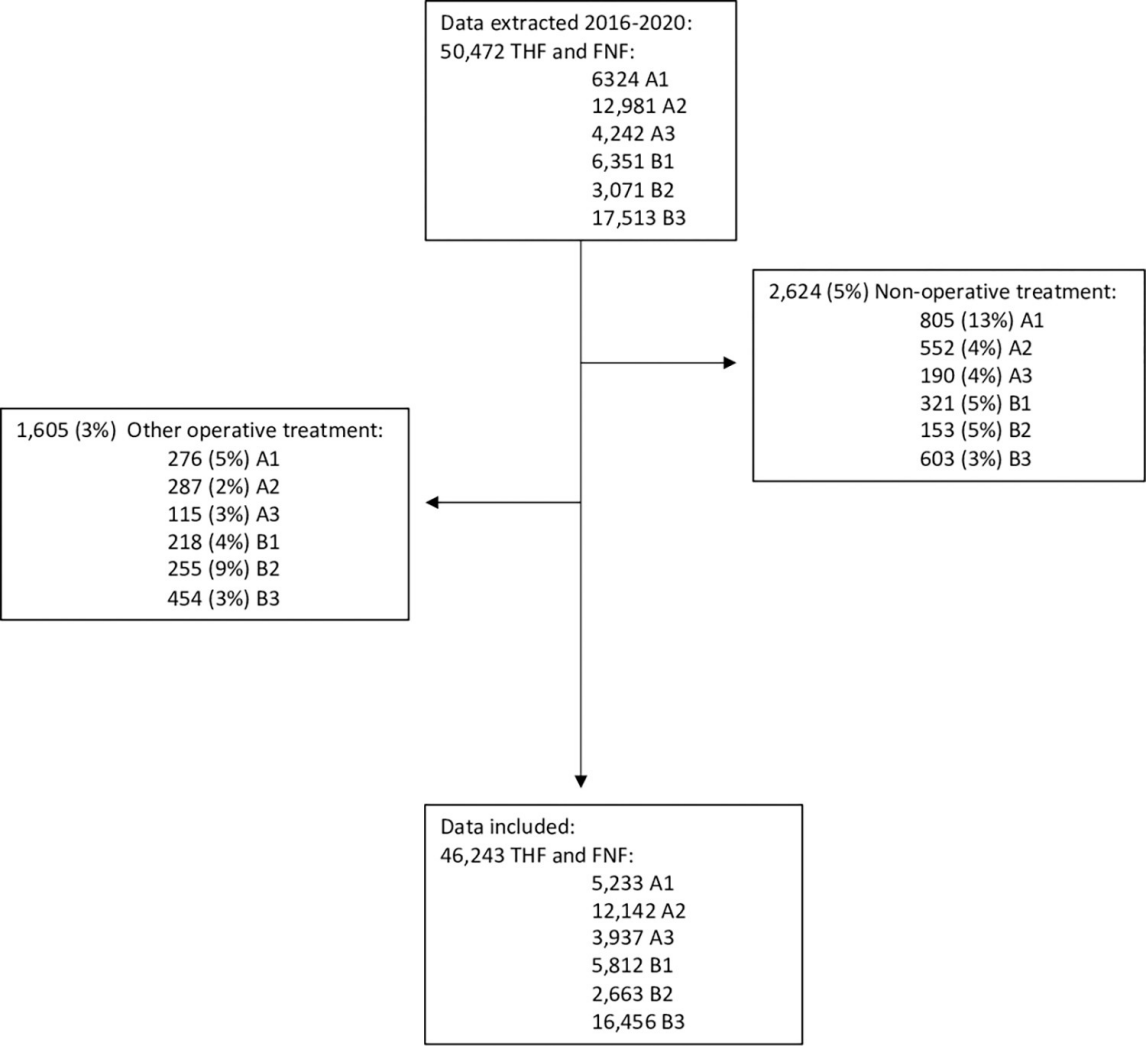
Flowchart showing patient selection. 46,243 patients ≥65 years registered in the SFR with a trochanteric hip fracture (THF) treated with a sliding hip device or an intramedullary nail or femoral neck fracture (FNF) treated with internal fixation or arthroplasty in 2016–2020.

**Fig 2:**
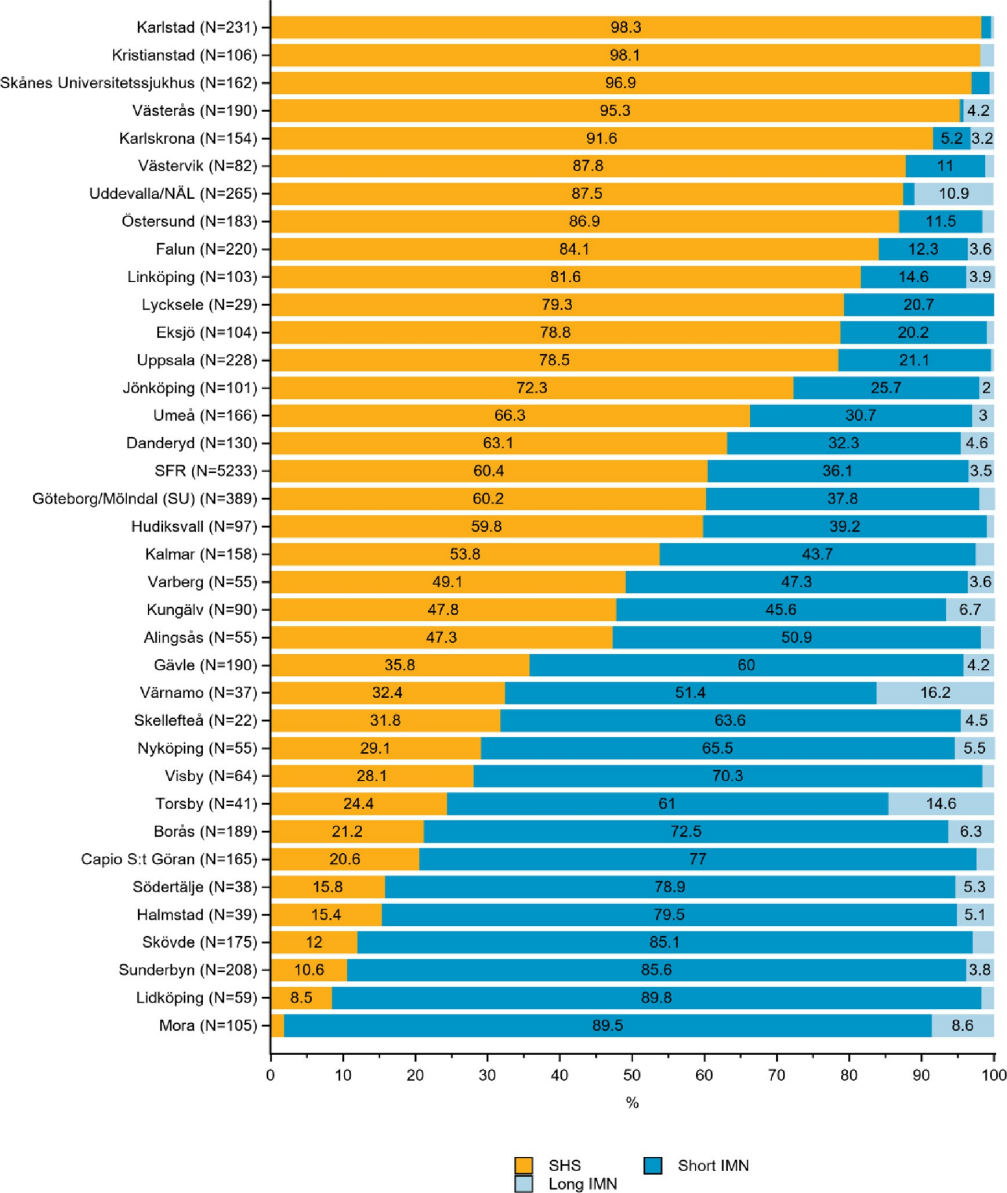
Distribution of sliding hip screw (SHS), short and long intramedullary nail (IMN) in the treatment of A1 trochanteric hip fractures by department in the Swedish Fracture Register in 2016–2020. SFR denotes all registrations in the Swedish Fracture Register (distributions are nationwide average).

**Fig 3:**
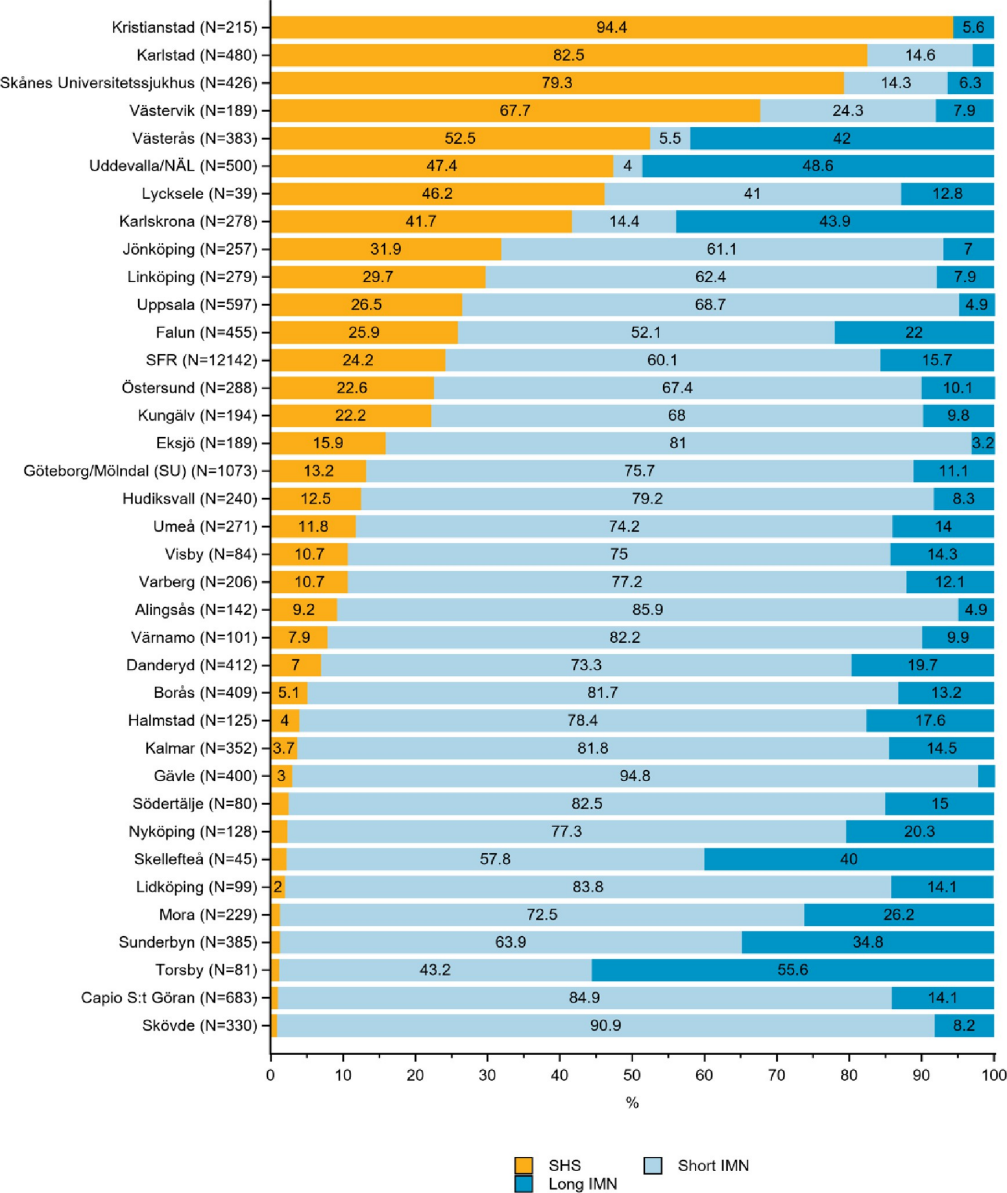
Distribution of sliding hip screw (SHS), short and long intramedullary nail (IMN) in the treatment of A2 trochanteric hip fractures by department in the Swedish Fracture Register in 2016–2020. SFR denotes all registrations in the Swedish Fracture Register (distributions are nationwide average).

**Fig 4:**
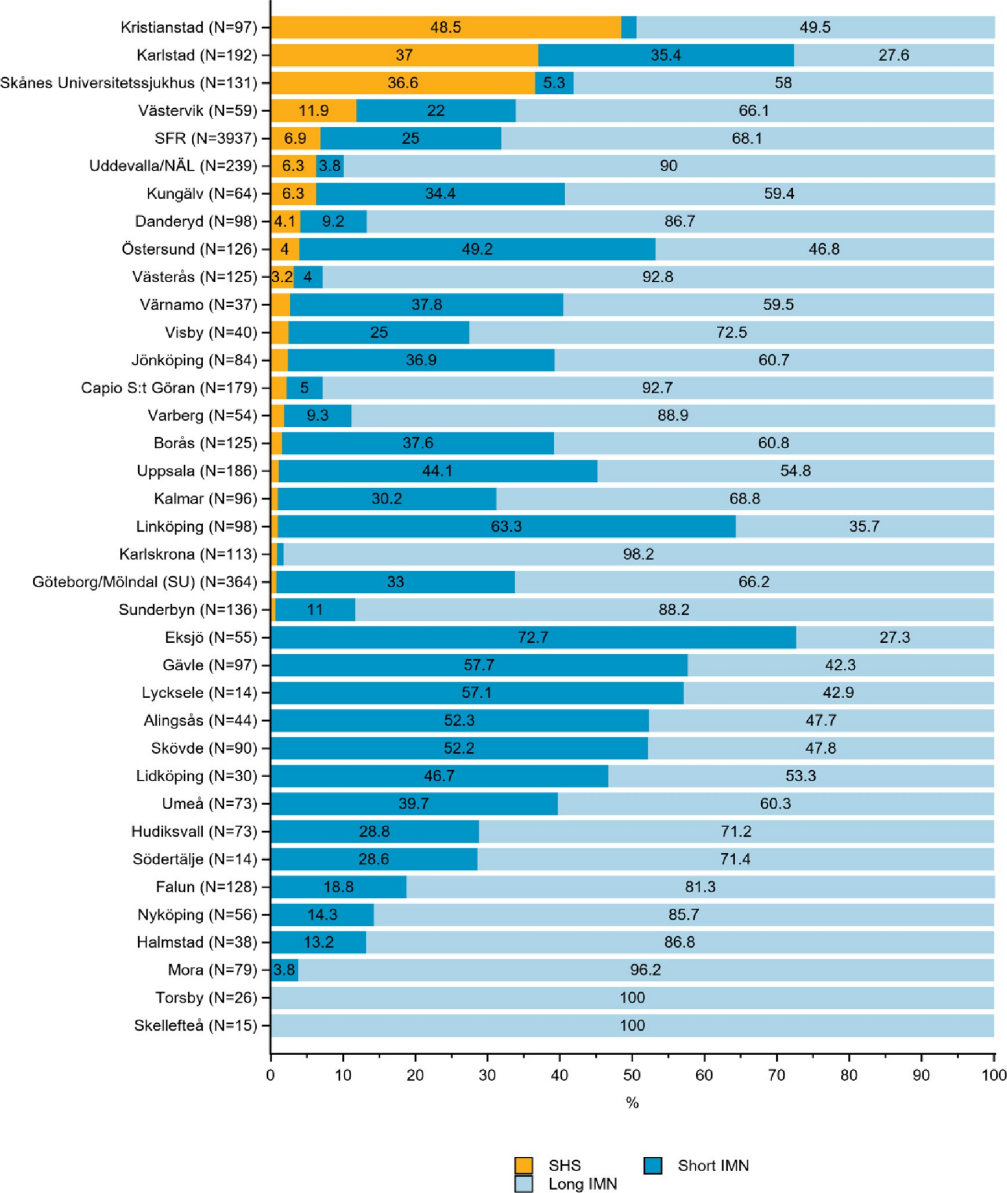
Distribution of sliding hip screw (SHS), short and long intramedullary nail (IMN) in the treatment of A3 trochanteric hip fractures by department in the Swedish Fracture Register in 2016–2020. SFR denotes all registrations in the Swedish Fracture Register (distributions are nationwide average).

**Fig 5:**
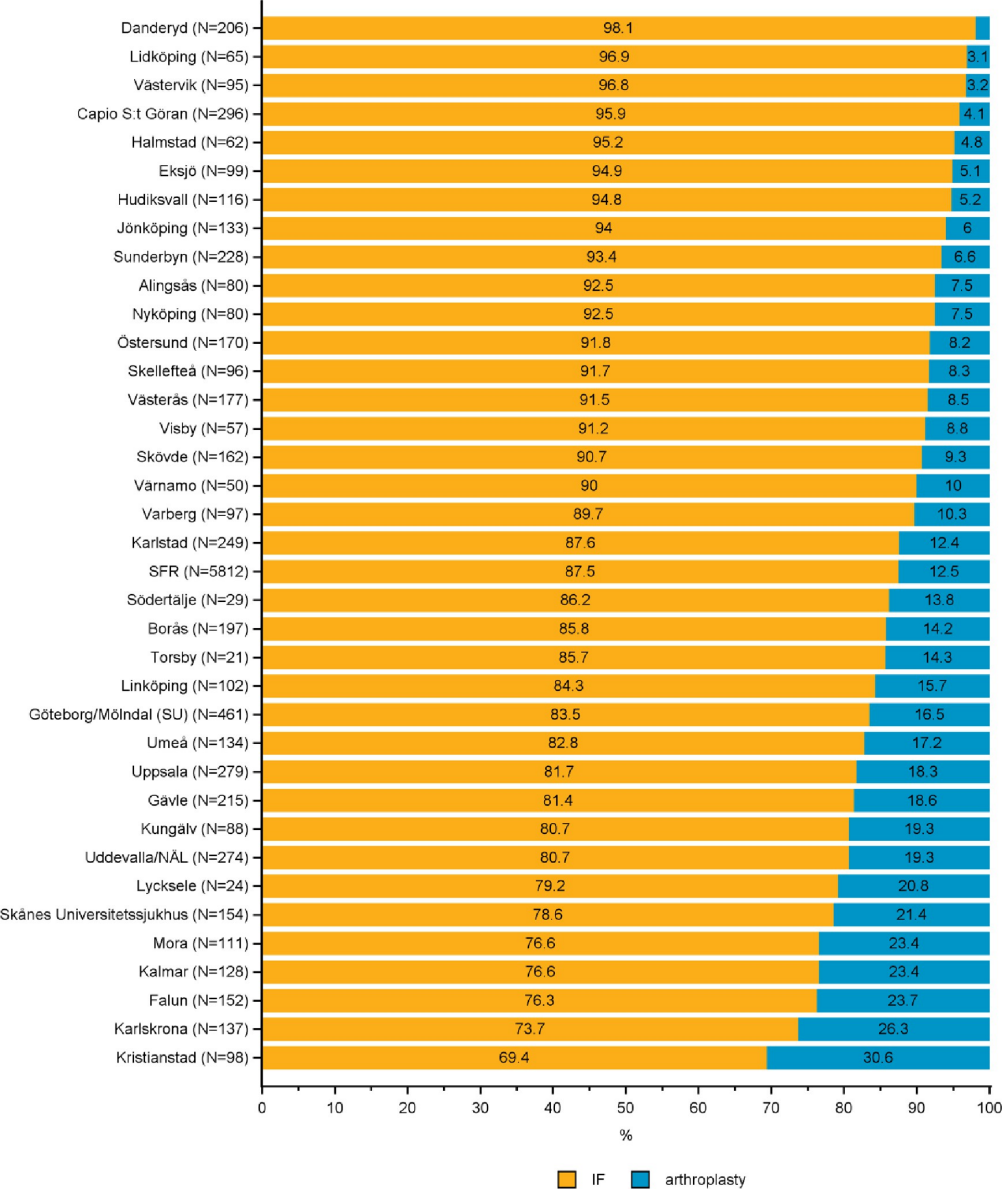
Distribution of internal fixation (IF) and arthroplasty in the treatment of B1 femoral neck fractures by department in the Swedish Fracture Register in 2016–2020. SFR denotes all registrations in the Swedish Fracture Register (distributions are nationwide average).

**Fig 6:**
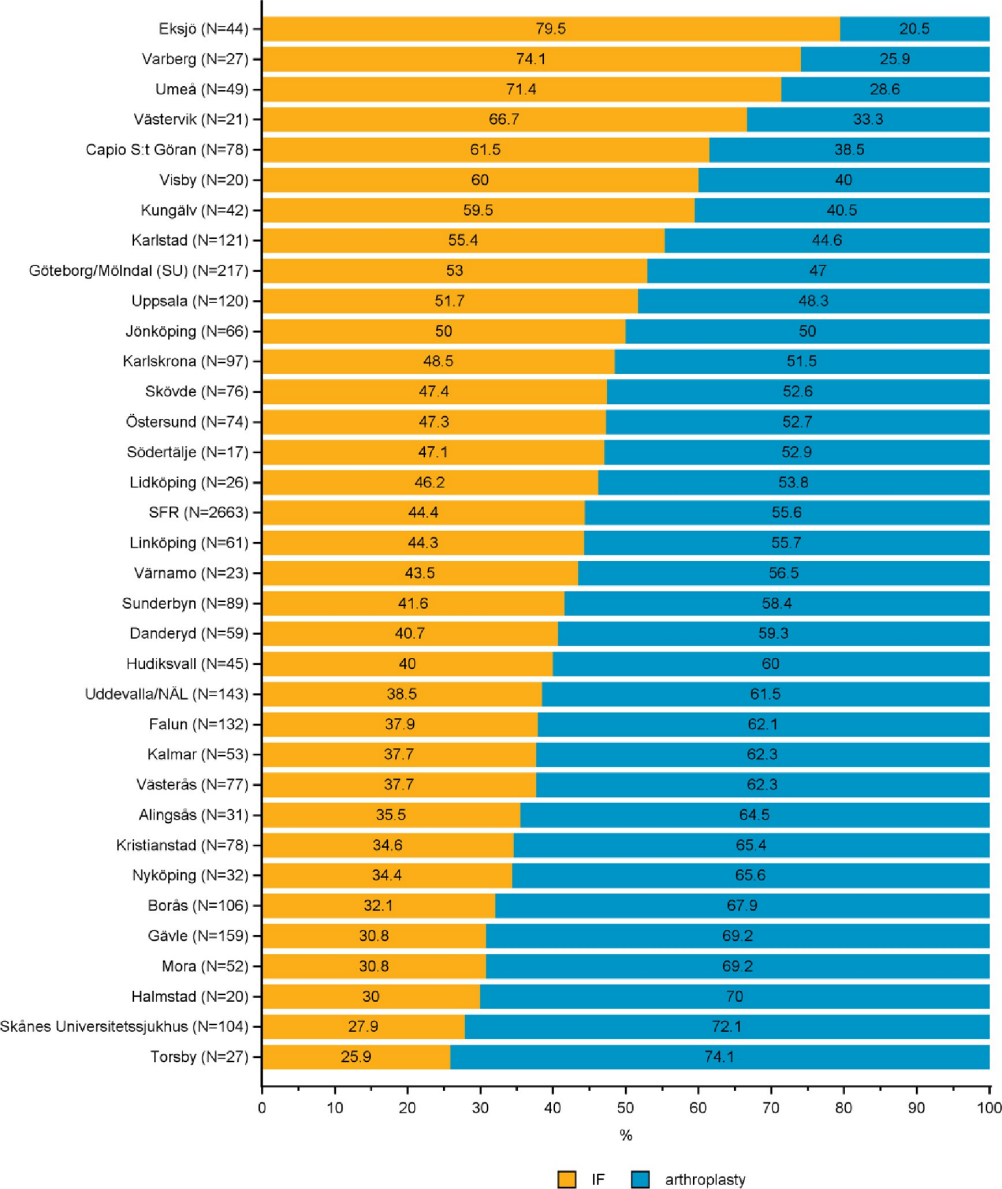
Distribution of internal fixation (IF) and arthroplasty in the treatment of B2 basicervical fractures by department in the Swedish Fracture Register in 2016–2020. SFR denotes all registrations in the Swedish Fracture Register (distributions are nationwide average).

**Fig 7:**
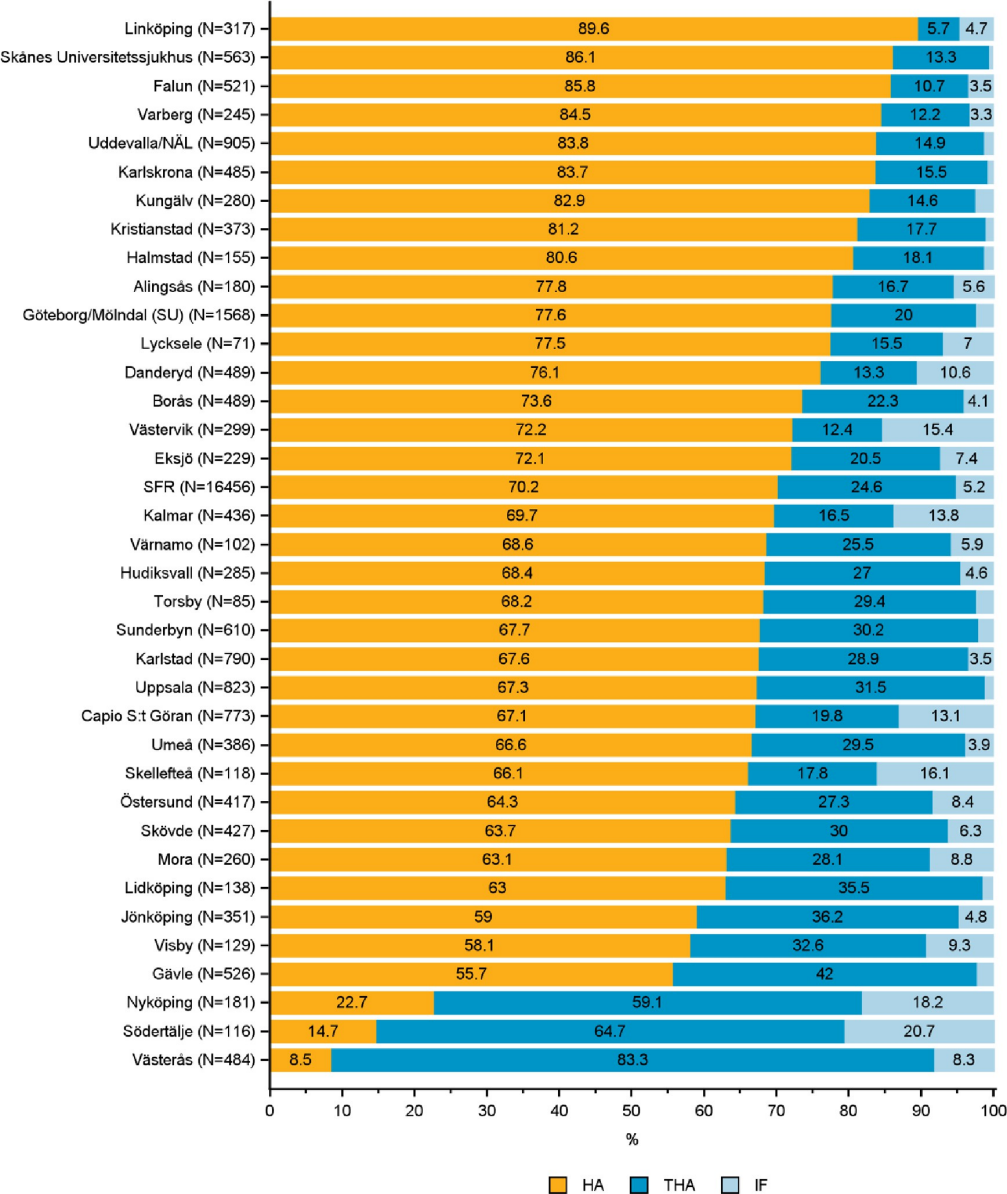
Distribution of hemiarthroplasty (HA), total hip arthroplasty (THA) and internal fixation (IF) in the treatment of B3 femoral neck fractures by department in the Swedish Fracture Register in 2016–2020. SFR denotes all registrations in the Swedish Fracture Register (distributions are nationwide average).

In this cohort study all patients ≥65 years at injury with a THF or an FNF registered in the SFR between 1 January 2016 to 9 September 2020 were included in the full study cohort (Fig 1, Flow chart, n = 50,472) (SFR in Figs 2–7). In addition to demographic details (age and sex), information on fracture types, treatment and treating hospital was retrieved.

Non-operatively treated patients were excluded (n = 2,624). We assessed the distribution of the main surgical procedures for each fracture type stratified by department, provided that completeness of ≥80% for femoral fractures was reached per department in 2020. For THFs, the inter-departmental variation between SHS, short IMN and long IMN was assessed and compared for A1, A2 and A3 fractures. For B1 and B2 fractures, variation between IF with pins, screws or SHSs and arthroplasty (HA or THA) was examined. For B3 fractures, variation between IF, HA and THA was compared. All other treatment options were excluded for the respective fracture types (Fig 1, n = 1,605).

### Ethics and data presentation

The study was approved by the Swedish Ethical Review Authority (reference number 2020–04662) and conducted according to the Helsinki Declaration. The dataset analysed in this study is not freely available because of legislation on register data and restrictions stipulated in the ethical permission to only report aggregated data. After obtaining ethical consent, data can be extracted from the Center of Registers, Västra Götaland. The study complied with the STROBE recommendations for observational studies [22].

### Statistics

Baseline epidemiological data are presented as number of patients, mean age (SD) and distribution (percentage) of the chosen operative treatment for each fracture type.

Inter-departmental treatment variation for the respective fracture types is depicted individually in graphs with department names (Figs 2–7) and compared to the nationwide treatment of that fracture type (SFR total).

## Results

46,243 hip fractures were registered in the SFR during the study period and were eligible to include in the study cohort.

### Trochanteric hip fractures

21,312 THFs were registered in the SFR. Distribution of fracture type, age, sex and treatment method is summarised for A1, A2, and A3 fractures in Table 1.

**Table 1:** Distribution of the 21,312 trochanteric hip fractures in the study cohort according to the AO/OTA classification. Number of fractures, mean age (SD), sex distribution (% women), treatment n (%) as sliding hip device (SHD) and short or long intramedullary nail (IMN).

AO/OTA	n	Age (SD)	Women	SHD	Short IMN	Long IMN
**A1**	5,233	84 (8)	65%	3,160 (60%)	1,891 (36%)	182 (3%)
**A2**	12,142	84 (8)	71%	2,941 (24%)	7,300 (60%)	1,901 (16%)
**A3**	3,937	83 (8)	72%	271 (7%)	984 (25%)	2,682 (68%)

### Inter-departmental variation

*AO/OTA type A1 –stable two-fragment fractures*. The most commonly chosen surgical method for stable two-fragment fractures was SHS, used in 60% of all cases (SFR, Fig 2). Treatment with SHS ranged from 2 to 98% between departments. IMN was used in 0 to 90% of the A1 fractures depending on the department (Fig 2).

*AO/OTA type A2 –unstable multi-fragmentary fractures*. A short IMN was used to treat most unstable multi-fragmentary fractures (employed in 60% of patients overall). SHS devices were the second most common, used in 24%, while 16% of patients received a long IMN (SFR, Fig 3). The use of a short IMN among departments varied from 4 to 94% in treating A2 fractures (Fig 3).

*AO/OTA type A3- reverse oblique or subtrochanteric fracture*. Overall, long IMNs were preferred to treat reverse oblique or subtrochanteric fractures, used in 68% of all cases (SFR, Fig 4). Proportions ranged from 27 to 100% among departments. Even though long IMNs were used in most cases, several departments used short IMNs for most of their A3 fractures. SHS was used to a lesser extent; however, in one department this approach was the treatment of choice for almost half of the A3 fractures (Fig 4).

### Femoral neck fractures

24,931 FNFs were registered in the SFR. Distribution of fracture type, age, sex and treatment method is summarised for B1, B2 and B3 fractures in Table 2.

**Table 2:** Distribution of the 24,931 femoral neck fractures in the study cohort according to the AO/OTA classification. Number of fractures, mean age (SD), sex distribution (% women), treatment n (%) of internal fixation (IF) and arthroplasty for B1 and B2 fractures; IF, hemi arthroplasty (HA) and total hip arthroplasty (THA) for B3 fractures.

AO/OTA	n	Age (SD)	Women	IF	Arthroplasty	
**B1**	5,812	82 (8)	68%	5,086 (88%)	726 (12%)	
**B2**	2,663	83 (8)	59%	1,182 (44%)	1,481 (56%)	
				**IF**	**HA**	**THA**
**B3**	16,456	83 (8)	64%	859 (5%)	11,552 (70%)	4045 (25%)

### Inter-departmental variation

*AO/OTA type B1* -*undisplaced or minimally displaced FNF*. IF was used in 88% of the 5,812 type B1 fractures in the cohort (SFR, Fig 5). Comparing departments, the proportion of IF varied between 70 and 98%. In some departments almost one third of the B1 fractures were treated with primary arthroplasty (Fig 5).

*AO/OTA type B2 –basicervical FNF*. Hip arthroplasty was used in 56% of the 2,663 type B2 fractures compared to IF in 44% (SFR total, Fig 6).

Arthroplasty use varied between 21 and 74% among departments (Fig 6).

*AO/OTA type B3—displaced FNF*. The cohort included 16,456 type B3 fractures. HA was used in 70% of patients and THA in 25% (SFR, Fig 7). Some 5% of the B3 fractures were treated with IF. HA use varied from 9 to 90% among departments. In all but three departments HA was performed in most B3 fractures. These three departments used THA in 59 to 83% of the fractures. IF use varied from 0.6 to over 20% of the B3 fractures in one department (Fig 7).

## Discussion

In this observational study on 46,243 hip fractures registered in the SFR we observed substantial inter-departmental variation for the chosen surgical method, regardless of fracture type. We can therefore determine that no nationwide consensus exists regarding surgical approaches to treat hip fractures.

To minimise unwarranted differences in hip fracture treatment between orthopaedic departments, decision making regarding hip fracture treatment should involve the best available scientific evidence and health economic considerations to achieve nationwide consistent care.

We believe that local traditions and “surgical signature” are important in explaining the discrepancies between departments. Traditions about selected surgical treatment are often passed on from tutor to trainee and are influenced by regional training and surgeon-specific beliefs, which could override established scientific evidence. Treatment decisions are often driven by social knowledge shared among colleagues who are influenced by meetings and conferences [23].

### Trochanteric hip fractures

During the first decade of the 21^st^ century, the use of IMN increased from 5 to 20% for THFs in Sweden [24]. Even for stable two-fragment THFs, there is a steadily growing use of IMNs [25, 26]. The NICE guidelines recommend using SHS over IMN for THFs [1]. Based on two observational studies from the UK and Sweden, IMNs are associated with a slightly increased short-term mortality [6, 7].

Despite this association, the frequency of IMN use is 25% for THFs in the UK, comparable to the distributions in our study (from 0 to 97%) [8]. These data suggest that guidelines alone do not change decisions about the surgical method. Several factors need to be in place to facilitate their implementation. We believe education, inter-departmental cooperation, reimbursement policies and continuous monitoring via a mature national quality register will be integral.

### Femoral neck fractures

A recent Cochrane report concluded that any benefit of THA compared to HA is likely to be small and not clinically significant [27]. The HEALTH trial found no short-term benefit of THA over HA for patients >50 years with a displaced FNF after a 2-year follow-up [28]. Not even the youngest (50–70 years) and fittest (ASA grade I or II) patients benefit from a THA with a short-term follow-up of 2 years [15]. However, the potential benefit of THA is the avoidance of acetabular erosion and worsening hip function with time, which can come into play in younger and active patients with a longer life span. With a higher age cut-off between IF and arthroplasty in Sweden, acetabular erosion has not been a clinical problem [29], as active ‘young old’ patients have been routinely treated with IF. The variation in practice we found is probably multifaceted. The individual experience and procedural volume of the surgeon at each department, combined with the choice of surgical approach and available implants, could influence the choice between HA and THA [30–32].

### National guidelines and treatment algorithms

Sweden was early (2003) to publish national guidelines [18]. National guidelines in the UK and the US have been available for at least 10 years [1, 2], aiming to provide a more standardised and evidence-based treatment for patients with hip fractures. Inferior implants have been discarded and cemented femoral stems are recommended to decrease the incidence of periprosthetic fractures [1].

Our finding of wide variation in the surgical treatment of hip fractures is similar to reports from the UK, where large inter-unit variation in SHS or IMN for THFs have been identified in combination with poor adherence to guidelines in using THA for hip fractures [8, 33]. Surgeon experience may play a part in explaining the variation seen in our study. This local surgeon experience is further inherited from mentor to pupil as fellowship experiences are not so common in Sweden, which maintains local treatment traditions or algorithms.

As a patient, you would expect similar and equivalent care, adhering to the best available evidence, regardless of the geographic location of the hip fracture; Our findings of large inter-departmental variation challenge these expectations. Many other factors need to be explored to understand to what degree these injuries are uniformly managed on a national level. These factors may include time from injury to surgery, factors related to inpatient care, access to rehabilitation, physiotherapy, walking aids and follow-up regimens. In addition, access to fracture-liaison services varies and has been linked to a reduced risk of future fractures [34].

Deviating from the recommended care standard may harm patient outcomes [35]. Thus, steps must be taken to disseminate knowledge on best practices and consequences of deviation and reduce the importance of surgeons’ personal preferences for treatment decisions.

Hospital reimbursement could facilitate policy changes and promote equal care [36]. The introduction of a Best Practice Tariff in the UK reduced pre-operative waiting time for surgery and 1-year mortality after a hip fracture [37]. Although some regions have reimbursement models to achieve prioritised care for hip fracture patients, there is no national model in place in Sweden.

Access to a mature national quality register, such as the SFR, will be prove vital in monitoring treatment methods, their outcome and to what extent guidelines are acknowledged.

### Strengths and limitations

The large cohort accommodates comparisons between all Swedish departments, ranging from smaller regional hospitals to large university hospitals. In the inter-departmental comparisons, we included all departments with ≥80% completeness of registrations, which we see as a high degree. Moreover, the fracture classification of the SFR enables us to compare fracture types traditionally treated differently as regards surgical procedures. This classification of femoral fractures in the SFR has been evaluated and considered satisfactory [21].

Limitations to our study include concerns that apply to all register-based studies, namely completeness, coverage, miscoding, transfer errors and underreporting. Missing data leads to exclusions of patients and loss of important information. Given our inclusion criteria, we believe the data quality suffices to assess treatment regimens across different orthopaedic departments and compare treatment for different fracture types.

### Future perspectives

Further studies should examine to what extent the variation identified in the treatment of hip fractures impacts outcomes (e.g., mortality, re-operations and patient-reported outcome). The sizable discrepancies in surgical methods indicate a need for updated national guidelines in Sweden, a work under development. A future follow-up study to compare treatment methods and their effect on patient outcomes is warranted when such guidelines have been implemented.

## Conclusion

The considerable inter-departmental variation in the surgical management of hip fractures in Sweden appears unwarranted based on available evidence, indicating an urgent need for updated national guidelines.
